# A Novel Orchestrator Architecture for Deploying Virtualized Services in Next-Generation IoT Computing Ecosystems

**DOI:** 10.3390/s25030718

**Published:** 2025-01-24

**Authors:** Francisco Mahedero Biot, Alejandro Fornes-Leal, Rafael Vaño, Raúl Reinosa Simón, Ignacio Lacalle, Carlos Guardiola, Carlos E. Palau

**Affiliations:** 1 Communications Department, Universitat Politècnica de València, 46022 Valencia, Spain; framabio@upv.es (F.M.B.); ravagar2@upv.es (R.V.); rreisim@upv.es (R.R.S.); iglaub@upv.es (I.L.); cpalau@dcom.upv.es (C.E.P.); 2 Thermal Engines Department, Universitat Politècnica de València, 46022 Valencia, Spain; carguaga@upv.es

**Keywords:** service orchestration, Cloud Native, edge–cloud continuum, Internet of Things

## Abstract

The Next-Generation IoT integrates diverse technological enablers, allowing the creation of advanced systems with increasingly complex requirements and maximizing the use of available IoT–edge–cloud resources. This paper introduces an orchestrator architecture for dynamic IoT scenarios, inspired by ETSI NFV MANO and Cloud Native principles, where distributed computing nodes often have unfixed and changing networking configurations. Unlike traditional approaches, this architecture also focuses on managing services across massively distributed mobile nodes, as demonstrated in the automotive use case presented. Apart from working as MANO framework, the proposed solution efficiently handles service lifecycle management in large fleets of vehicles without relying on public or static IP addresses for connectivity. Its modular, microservices-based approach ensures adaptability to emerging trends like Edge Native, WebAssembly and RISC-V, positioning it as a forward-looking innovation for IoT ecosystems.

## 1. Introduction

### 1.1. General Background

Cloud Native is a recent and increasingly adopted paradigm that emerged for designing, deploying and managing applications in cloud computing environments. It is based on different pillars, such as the decomposition of applications into microservices, the use of containers as virtualization technology, and the automation of the development and operation phases (DevOps). As a result [1], properties such as scalability, failure resilience and continuous operation (even during upgrades) can be achieved. The key technology of this paradigm is Kubernetes (K8s) [2], a widely known open-source platform for managing containerized workloads and services, facilitating their configuration and automation.

Although originally conceived for cloud environments, the core concepts of Cloud Native can be adapted to edge environments and, therefore, IoT applications and services can benefit from it. The flexibility brought by this paradigm is crucial for the evolution of IoT, the so-called Next-Generation IoT (NGIoT). According to [3], this evolution comes with a set of key technological enablers such as edge computing, 5G, Artificial Intelligence (AI), advanced analytics, augmented reality, digital twin and Distributed Ledger Technologies (DLT), which may be present in an IoT system to address use cases with complex requirements (e.g., with low latency needs, real-time human interaction, advanced processing, intelligence based on business logic, auditing, etc.). Since these enablers are technologically decoupled and can evolve at different paces, the adoption of Cloud Native concepts for NGIoT is crucial for easing their integration, upgrade and removal.

Depending on its scope, an NGIoT system can be supported by IoT, edge, fog and cloud infrastructure—the IoT–edge–cloud computing continuum. Resource virtualization is an essential enabler for the continuum, as it allows abstracting and isolating the underlying physical resources thus easing the deployment of services and applications on top of them. However, combining these ecosystems is not trivial and some challenges need to be overcome [4,5]. In contrast to cloud, a set of key features have to be fulfilled for taking advantage of the computing continuum, including heterogeneity and interoperability of physical and virtual devices, real-time support, location awareness, dynamicity (i.e., resources being included/removed, with varying networking configurations and processing capabilities) and wireless communication schemas. In addition, as services might end up scattered within delocalized computing nodes, security becomes an even more challenging aspect, as the number of attack surfaces increases.

**Figure 1:**
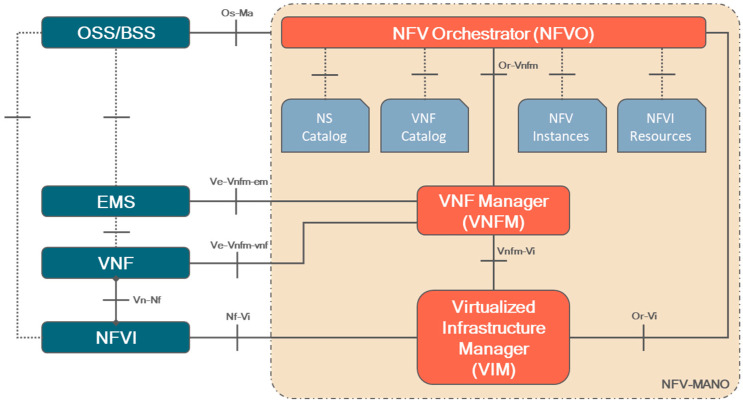
ETSI MANO components and interfaces.

Orchestrators are thus needed to manage both the virtualized infrastructure and the services lifecycle. Currently, there are studies and solutions partially covering the mentioned aspects. One orchestration architecture worth presenting is the Management and Orchestration (MANO) standard for Network Function Virtualization (NFV) [6], defined by the European Telecommunications Standards Institute (ETSI). As one can see in Figure 1, it consists of three main functional blocks: the NFV Orchestrator (NFVO), which is responsible of onboarding Network Service (NS) and Virtualized Network Function (VNF) packages, managing the lifecycle management of NS and global resource management; the VNF Manager (VNFM), in charge of the managing the lifecycle of the VNFs of the deployed NSs (from instantiation to scaling, healing, modification and termination operations); and the Virtualized Infrastructure Manager (VIM), which controls and manages the virtualized computing, storage and networking resources.

This standard is leveraged by existing NFV orchestrators such as (i) Open Source MANO (OSM) [7], the framework developed under the ETSI umbrella, which supports both VM-based (over OpenStack, AWS, etc.) and container-based (over K8s) VNFs, with more features implemented for the first type. In OSM, the user has full deployment control, as no intelligence is added for allocating the services. Developed by OpenStack, (ii) Tacker [8] has container-based support for VNFs (over K8s), although many transversal aspects have to be managed by the users. From the Linux Foundation (LF), (iii) ONAP [9] is a platform for orchestrating, managing and automating network and edge services. It includes more features than the former solutions (e.g., service design and creation, external systems integration, data collection and analytics, visualization, security and networking tools, etc.), being thus more complex. With the proper descriptors for deploying the applications, VNFs can be deployed over K8s, OpenStack or any commercial VIM, including policy-driven intelligence for workloads placement/scheduling. Also under the umbrella of the LF, (iv) Anuket [10] delves deeper into the MANO part for both VM-based (over OpenStack) and container-based (over K8s) VNFs, also introducing concepts from GSMA’s reference model [11]. Finally, although not MANO-compliant, (v) EMCO [12] (now also a LF project) is one of the more complete solutions for containerized VNFs, developed specifically for K8s (but yet supporting VM-based workloads). It includes different plugins for managing several K8s aspects, including multicluster environments, and follows an intent-driven model for automatically selecting the optimal placement of services on the continuum. Still, most of these orchestrators are considered only for the telco ecosystem, with powerful yet rather homogeneous computing capabilities, not fully exploiting the heterogeneity of the available resources.

Apart from NFV orchestration architectures, designed for the specific requirements and constraints of network services, other orchestration architectures are available. Orive et al. [13] presented an architecture for the edge–cloud continuum, based on K8s and accompanied by a set of information models for the infrastructure and the workloads, and considering different QoS-based optimization criteria. In [14], the authors followed a similar approach, but considered a multicluster K8s environment. Other approaches extend resource orchestration with Software Defined Networking (SDN) to optimize as well the networking paths, as in [15], although in this latter case for Virtual Machines (VMs) instead of containerized-based workloads. Also, different European actions have been advancing toward the management of heterogeneous environments, the implementation of scheduling and allocation intelligence, and the execution of business applications (i.e., not just VNFs). Starting with the SERRANO platform [16], it automates the process of applications deployment (designed with its own SDK) across various computing technologies, translating their high-level requirements to infrastructure-aware configuration parameters. It works for containers, VMs and Unikernels, using its own abstraction layer for leveraging the underlying computation and storage resources. In contrast to the former, RAINBOW [17] provides compatibility with existing container orchestrator platforms (i.e., Helm charts, Docker compose) but only works with containers (not VMs or Unikernels). It provides dedicated tools for easing the deployments (with sophisticated intelligence for resource provisioning) and communication of services. The greater contribution of this orchestrator lies on its policies, which can dictate not just installation but also operation behavior. Continuing with the ACCORDION platform [18], it also focuses on managing heterogeneous computing nodes of the edge–cloud continuum, targeting containers, VMs (via KubeVirt integration) and Unikernels, all on top of K8s clusters (specifically, K3s). While all these platforms support security, telemetry and analysis features, the latter is the only one that considers MANO specifications for deploying VNFs.

Fleet [19] should be also highlighted as a scalability engine focused on Kubernetes deployments. This solution, based on custom K8s resource definitions, is both a cluster engine and a deployment manager for Kubernetes, allowing configuring a large volume of clusters from a single, central point. It supports managing deployments with raw Kubernetes manifests, Helm charts or Kustomize technologies, although it does not implement service lifecycle control nor includes any kind of placement intelligence as do the previous solutions.

These orchestration architectures and solutions do not address the management of computing resources with dynamic, changing IP configurations, a critical aspect in many IoT–edge scenarios. Among open-source platforms that consider this aspect, one can highlight KubeEdge [20], KubeVela [21] and OpenYurt [22], each of them offering unique capabilities. KubeEdge extends K8s to the edge, enabling efficient edge–cloud collaboration by synchronizing workloads and managing edge nodes even in intermittent network conditions. OpenYurt builds on native Kubernetes, enhancing it with edge-specific functionalities such as autonomous edge operation and graceful node recovery, without compromising cloud compatibility. Finally, KubeVela acts as an application delivery and management platform, enabling developers to define and manage edge workloads with declarative application-centric abstractions.

### 1.2. Motivation and Objectives

To address the aforementioned challenges, a dedicated NGIoT orchestrator architecture is needed to manage the lifecycle of services in distributed, heterogeneous and dynamic computing environments. This architecture must consider features such as AI-supported service allocation, secured communication, fault tolerance, dynamic resource management and full observability of the involved resources and services. While many open and commercial solutions can be found for managing resources in cloud environments (with Amazon Web Services, Microsoft Azure and Google Cloud taking over most of the market), orchestrators that span across the whole continuum are not that mature.

The aim of this study is to present the design and development of a resource and service orchestrator for the IoT–edge–cloud continuum, focusing on Cloud Native, security, scalability and dynamicity aspects for addressing the features demanded by NGIoT applications. It supports network-related (i.e., VNFs, compliant with ETSI MANO) and non-network related applications, VM and containerized-based workloads, and allows the use of different algorithms for service allocation. While there are common features with existing propositions and solutions, the main novelty lies in the management of dynamic computing nodes. In this article, dynamicity is understood as a feature that enables the utilization of edge and IoT computing nodes that are not placed in specific, fixed locations, which implies that (i) connectivity is generally wireless, leveraging technologies such as WiFi and 5G; (ii) connectivity might be lost, when there is no coverage between access points/base stations and the managed resources; (iii) more than one radio technology might be available for managing them, and involved IP addresses might be dynamic; and (iv) deployment and runtime errors are more likely. The proposed orchestrator architecture has been designed, implemented and validated in the four real-world pilots of the H2020 ASSIST-IoT project.

The remainder of this paper is organized as follows: In Section 2, the design and a technological implementation of the proposed orchestrator is presented. The utilization and performance of the solution in the context of a challenging automotive NGIoT scenario are described in Section 3, along with the experimental setup deployed. A discussion about the solution, existing alternatives and future evolution is provided in Section 4. Finally, conclusions are drawn in Section 5.

## 2. Materials and Methods

### 2.1. Orchestrator Architecture

**Figure 2:**
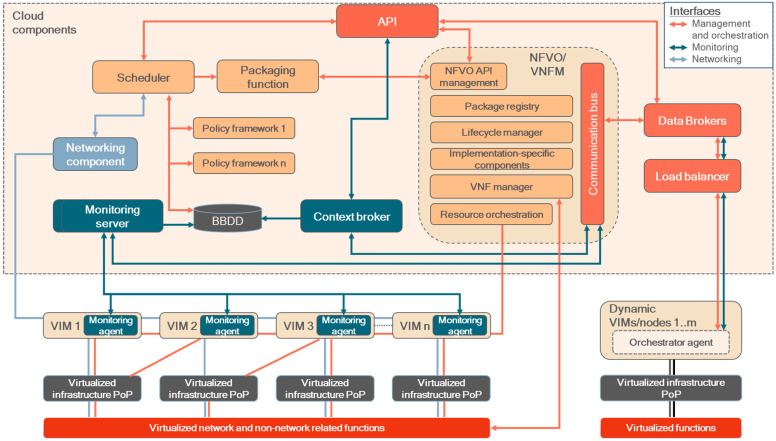
High-level architecture of the proposed orchestrator. The cloud components and the orchestrator agent are the core building blocks of the solution. VIMs, virtualized infrastructure PoPs, virtualized network and non-network functions are external components defined in MANO.

The orchestrator’s architecture is depicted in Figure 2. It complements and extends the ETSI MANO framework by (i) implementing intelligent service scheduling capabilities, (ii) integrating Cloud Native features related to networking and monitoring, and (iii) supporting both massive and dynamic configurations. To enable the latter, a design based on a publish–subscribe pattern is considered, where the managed VIMs (expected to be mobile and/or temporal computing nodes) host an agent that will grant communication with the orchestrator, ensuring error handling in case of loss of connectivity or reconfiguration, integrating dedicated functions to support local lifecycle management of services. To differentiate these VIMs from those typically managed by MANO (with fixed, public IP connectivity), they are hereafter referred as “dynamic VIMs”. This design considers the following features:

1. A hierarchic pattern, where groups are defined and then dynamic VIMs are registered and belong to them. All the VIMs of a specific group will host the same set of services, yet with the possibility of instantiating specific services in a particular VIM.
2. Services are locally installed, updated or deleted depending on the messages received in the agent from the orchestrator. A set of components are devoted to these processes, from the download of the containers images (asynchronous) to the deployment and deletion of services (minimal version of NFVO/VNFM).
3. In the case that the orchestrator’s agent cannot recover from errors, these are communicated to the cloud via dedicated topics, as communication might be lost during package downloading, service installation or messaging.
4. Messages should be transmitted in a secured way (e.g., integrating TLS, access control mechanisms, etc.) to ensure that malignant actors cannot deploy malignant services or disrupt the expected operation of the system.

This design supports massive deployments where a common set of services are expected to be deployed independently in large groups of nodes or clusters; with just a single command, a specific service can be deployed, updated and deleted in all of them. Being more scalable than a server–client design, the key benefit is the possibility of managing temporary nodes or clusters (with minimal installation) and those in which fixed, public IP addresses cannot be ensured/provisioned, as having an Internet connection is enough.

A brief description of the functionalities of the main building blocks of the orchestrator architecture is now presented, including the typical MANO features extended with a scheduler for service allocation, networking and monitoring capabilities:

- API: It is the main entry-point of the solution, from which a user (directly or through graphical interface) can access the features of the orchestrator, including those related to dynamic VIMs.
- NFVO/VNFM: This module groups ETSI MANO components, from the registration/update/removal of VIMs and service repositories to the lifecycle management of services. As its traffic can increase exponentially if several VIMs and services are managed, a communication bus is implemented.
- Scheduler: This component acts when a user lets the orchestration decide the placement of a service to be deployed. Regarding the policy frameworks, different models and tools can be considered.
- Function packaging: It prepares the NS and VNF descriptors as demanded by MANO specifications, gathering the required data from the services’ manifests.
- Networking component: It communicates with the VIMs to implement layer 3 networking rules related to security, to allow/prevent communication between specific components of the managed services.
- Monitoring server: This component handles, homogenizes, aggregates and stores different metrics from all the managed VIMs and deployed services. Data will come from the monitoring agents deployed in each of the VIMs.
- Internal database (BBDD): It hosts relevant data, from the metrics collected by the monitoring server, to the data needed/generated by the policy frameworks (training data, AI models) and information related to the dynamic VIMs.

In the architecture, one can observe different connections among Virtualized Infrastructure Points of Presence (PoPs) and VIMs. They represent that the abstracted resources of a given PoP (e.g., a virtualized server or edge node) could be controlled by one or many VIMs (e.g., Kubernetes clusters, OpenStack), and that one VIM may control one or many PoPs. Also, the communications among services within or among VIMs are governed by the rules applied by the networking component.

**Figure 3:**
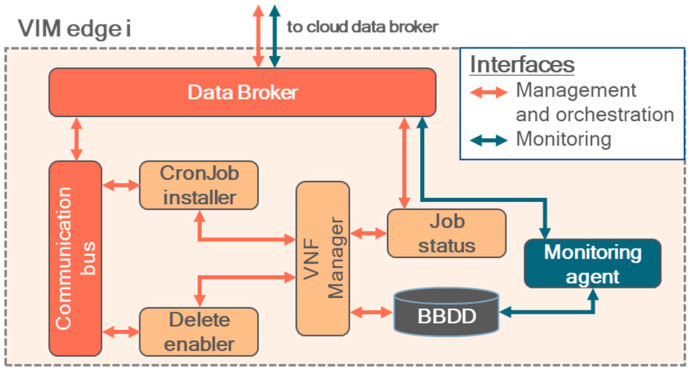
Component decomposition of the orchestrator agent. Communication with the cloud counterpart of the orchestrator is managed by the data brokers, with messages through the pub–sub topics presented in Section 2.2.

Now, the building blocks for supporting dynamic VIMs are presented. The involved components provide support for use cases where VIMs cannot be operated with MANO considering a typical flow, due to technological or operational restrictions:

- Data brokers: They handle the exchange of data between the agents of the dynamic VIMs and the orchestrator (one instance on each). MQTT, AMQP or similar transport protocols could be implemented. Distributed instances are recommended to enhance the availability and scalability of dynamic clusters.
- Load balancer: Distributes the load of messages coming from the orchestrator agents between the available data broker instances of the orchestrator.
- Communication bus: Besides supporting NFVO/VNFM messages, it enforces the operation of the data brokers to ensure that data are not lost when connectivity is not available (i.e., fault tolerance mechanisms).
- Context broker: This component handles the status of the dynamic VIMs, including the status of the services deployed on them and error-related data.
- Orchestrator agent (Figure 3): Deployed on each dynamic VIM, it has two main purposes: governing the local lifecycle of the virtualized services (similarly to a local NFVO/VNFM) and managing the communication of data with the orchestrator. To implement the latter, a local data broker and a fault-tolerance service are part of the agent, supported by a database. When use case-related data needs to be transmitted, the agent’s data broker could be considered for that end (API REST could also be considered).

A basic data model for managing the dynamic VIMs is depicted in Table 1. It includes three entities, namely group (where services can be deployed), dynamic VIMs (which must belong to one group) and service (deployed in a specific cluster, or dynamic VIM). The actual data are available at the context broker of the orchestrator.

### 2.2. Communication Interfaces

The orchestrator is expected to be operated through an API or a Graphical User Interface (GUI). A basic API for managing the lifecycle of Cloud Native applications has been designed, allowing for (i) registering and deleting VIMs (and groups of dynamic VIMs) to manage; (ii) registering, updating and deleting package repositories of services; and (iii) instantiating, upgrading and deleting services in the managed VIMs (manually specifying the target computing node or cluster or letting the scheduler component decide), dynamic VIMs and groups of them. Apart from managing the lifecycle of services, the API communicates with the networking policies to increase the security of deployments and with the scheduler, the latter only in case of automated service deployments.

To keep compatibility with ETSI MANO, a packaging component is included, which goal is to prepare the NS and VNF descriptors to facilitate interaction with legacy NFVO. In the Cloud Native paradigm, existing packaging technologies are Kustomize [23], Juju bundles [24] and Helm charts [25], the last being the de facto standard for providing production-ready packages. Thus, to prepare these descriptors, key information is extracted from the Kubernetes templates or manifests included in those packages.

Furthermore, when considering dynamic VIMs, the API calls related to the lifecycle management of services and their monitoring are translated to a set of pub–sub messages with predefined topics, exchanged between the data brokers of the orchestrator and the agents. A set of basic topics has been defined to that end (see Table 2). This design also comes with management advantages, as dynamic VIMs can register by themselves (with proper certificates) and services can be deployed in several of them with a single command, specifying the target group.

### 2.3. Scheduler and Policies

There are several works devoted to manage and distribute services within a managed infrastructure; however, most have been designed for centralized ecosystems [26], hence not suitable for computing continuum ecosystems in which resources might be geographically scattered. Still, for VMs, containers or both, there are some works targeting solutions for scheduling services in the optimal VIM based on latency/location awareness [26,27,28], burst forecast [29], available resources [30] or cost efficiency [31].

The proposed architecture foresees the possibility of having a scheduler with several policies/intents, to be selected by the user when deploying a service. These policies may be supported by different AI frameworks and leverage different monitoring metrics to operate, which can include reallocation capabilities. They can be related to QoS, optimization of managed resources and/or specific needs of resources, e.g., large storage or acceleration capabilities. Focusing on Cloud Native, specific Kubernetes objects such as horizontal or vertical autoscaling or resource requests could be modified by the outcomes of these policies to meet users’ intents.

### 2.4. Networking Policies

Security is a crucial factor for the success of any system, being in this case critical due to the large number of attack surfaces that may exist. There are several layers in which security can be tackled, at network (layer 3), transport (layer 4), application and access levels (layer 7). The latter layer is outside the scope of this section, as it involves software-specific implementation and services (e.g., API gateways, identity managers, authorization servers, etc.) that are beyond the functional features of the orchestrator, but necessary to secure its API.

The orchestrator architecture depicts a component that interfaces with the underlying VIMs’ network managers to allow or prevent communication among deployed services. For instance, traffic between services and applications could be limited to exposed interfaces, so that their internal microservices can only communicate among them and not with those from others. In the Cloud Native paradigm, these policies are governed by a specific K8s’ CNI plugin, which policies can be based on the presence of specific labels or annotations.

Apart from layer 3 rules, the CNI plugin is set to provide encryption for data traveling among computing nodes and clusters, hence providing transport security. Data from services belonging to the same computing node do not need to be encrypted, as raw traffic could be observed on the node anyway. Lastly, regarding the communication between data brokers, certificates will need to be issued so data do not travel unencrypted through public, unmanaged networks. Otherwise, the load balancer should deny the connections, with the TLS termination happening at the load balancer instead of the broker, thus relieving the broker of this task and reducing its workload.

### 2.5. Architecture Implementation

This section presents the set of technologies leveraged to develop an actual orchestrator implementation, considering mature open-source technologies (see Table 3). The code can be consulted from the public repository of the ASSIST-IoT project [32].

## 3. Results

This section delves into the operation and performance of the developed orchestrator in the framework of an exemplifying use case from the automotive sector. Specifically, this section presents (a) the target use case and involved devices; (b) the experimental setup used for validation; (c) an example of usage of the orchestration system, via graphical interfaces, to deploy the services involved in the use case; and (d) an evaluation of the performance of the solution, considering latency, scalability and reliability aspects.

### 3.1. Considered Use Case

In the automotive sector, regulation over emissions has driven the evolution of propulsion systems. Real-life emission compliance certification must be measured and ensured, not just before vehicles are released but continuously monitored once on the road, as mandated by the upcoming Euro 7 standard [36]. To that end, vehicles should be equipped with appropriate sensors, and the captured data should be processed locally, aggregated and judiciously sent to cloud for further processing (e.g., for alerting particular issues, for obtaining insights related to the entire fleet, for training machine learning models or combining models by using federated learning techniques, etc.) [37]. In this use case, and the sensors from which data are collected, vehicles must be equipped with a computing node with enough resources and wireless network interfaces (e.g., 4G, 5G).

This use case exemplifies several benefits of the proposed orchestration architecture. On the one hand, the architecture considers the proper mechanisms to manage a large number of mobile edge nodes, with dedicated connection-recovery and fault-tolerance mechanisms. On the other hand, it enables the seamless instantiation and automated monitoring of a common set of services in all of them. It should be mentioned that despite the fact that the orchestrator can manage the lifecycle of the cloud services for the use case, the big data architecture design (e.g., data warehouse/lake, batch processing, etc.) required to address the use case falls outside the scope of this paper.

### 3.2. Experimental Setup

**Figure 4:**
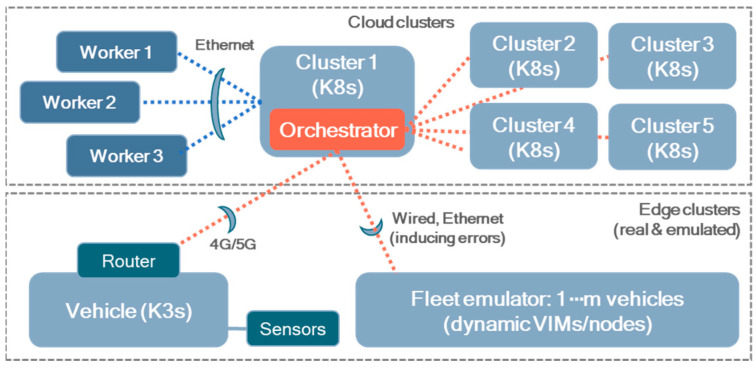
Computing infrastructure used for validating the use case. Dashed orange lines refer to VIMs registered and connected to the orchestrator, while blue ones symbolize the worker nodes of Cluster 1, where the cloud components of the orchestrator are deployed.

An experimental setup was prepared to deploy the implemented orchestrator and evaluate its capabilities in the framework of the considered use case. This setup integrates centralized cloud computing with edge computing and IoT to explore the interplay between local processing and centralized analytics. Particularly, the study utilizes a self-hosted, multicluster Kubernetes-based infrastructure deployed on virtual machines managed via Proxmox. The details of the computing infrastructure considered are summarized in Table 4 and depicted in Figure 4.

#### 3.2.1. Real-Time Vehicle Experiments

**Figure 5:**
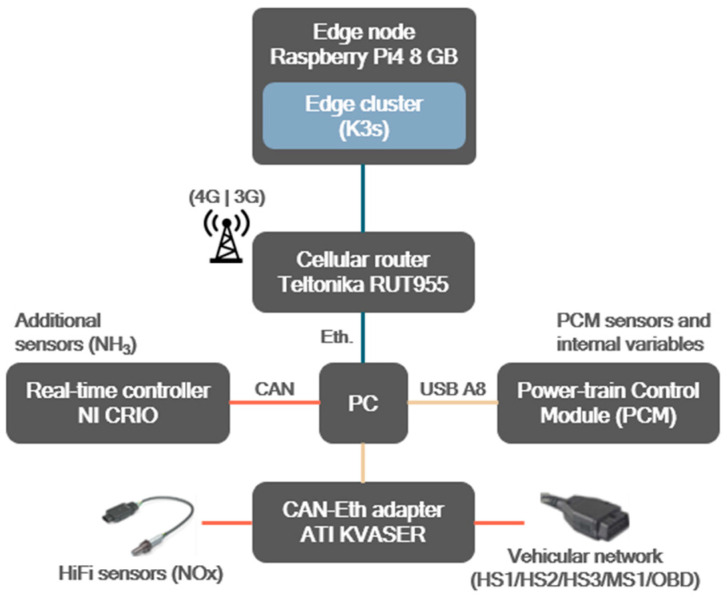
IoT–edge infrastructure deployed on the vehicle used in the use case.

Experiments were conducted using an actual car as part of the setup. This vehicle includes the sensors and devices presented in Figure 5, connected with different wired technologies (USB, Ethernet, CAN bus). In particular, it integrates (i) telemetry and diagnostic data from the vehicle’s Power-train Control Module (PCM); (ii) additional sensors to augment the vehicle’s native data collection, including environmental sensors (NOx, CO_2_, GPS); (iii) a local PC with Windows as required by ATI VISION, a legacy application needed to gather and process signals from the vehicle and transform them into valuable data; (iv) a Raspberry Pi4 that hosts a K3s cluster; and (v) a router providing cellular connectivity. More information can be found in [37]. This setup provided real-time data streams for validation of the system’s performance under operational conditions.

#### 3.2.2. Emulated-Based Experiments

To validate the scalability of the presented architecture, a dedicated cluster (“Fleet emulator”) was prepared to emulate the communication with a large fleet of vehicles. It was leveraged to perform most of the evaluation tests described in Section 3.4. The involved tests considered POSTMAN and JMeter to simulate API requests and evaluate system performance under controlled conditions. On the one hand, POSTMAN was used for functional testing of APIs, ensuring correct communication between components. On the other hand, JMeter facilitated stress and load testing to assess the system’s reliability and scalability under high-demand scenarios. This cluster was designed to emulate the connectivity of a fleet of >100,000 vehicles.

### 3.3. Orchestration Installation and Operation

**Figure 6:**
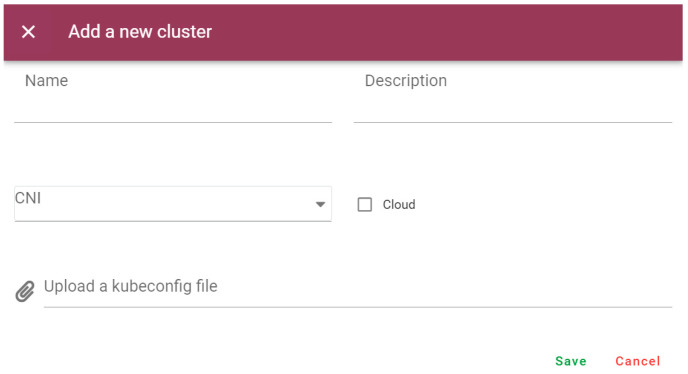
Graphical interface for registering a cluster. A similar interface is available for defining groups of dynamic VIMs.

The cloud components of the orchestrator were instantiated on “Cluster 1”, following the available documentation and packages [32]. Once running, the K8s’ cloud clusters (“Clusters 1–5”) and the group that includes the vehicle’s dynamic VIM are registered to the orchestrator, using the dedicated GUI interfaces (in Figure 6, for static VIMs). With the group defined, an instance of the orchestrator agent is deployed on the vehicle’s K3s. Then, its data broker can subscribe itself to the relevant topics, pointing to the IP address of the orchestrator and having valid SSL-TLS certificates.

**Figure 7:**
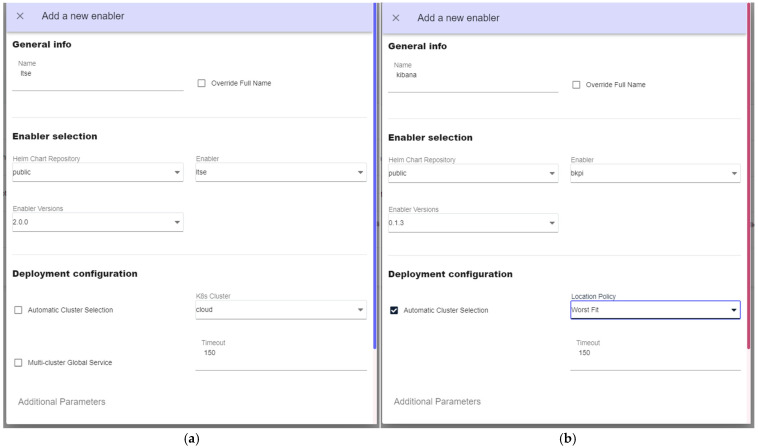
Graphical interfaces for deploying services: (**a**) manual cluster selection and (**b**) automatic selection based on scheduling policies.

Once the VIMs that host the use case services are registered, the following services are installed via the graphical user management interfaces: at cloud, (i) ASSIST-IoT’s LTSE (a service for SQL and NoSQL data storage, based on PostgreSQL and Elasticsearch, respectively) for storing use case specific data, (ii) Kibana, to represent NoSQL data from LTSE; at the edge, (iii) a preprocessing function for the gathered sensor data, (iv) an emission modeling function, (v) a sensor modeling function, a (vi) diagnostic function, and (vii) a NoSQL local database (MongoDB). The orchestrator offers different installation options. In this use case, the following ones are considered for the cloud-related services:

- Manual cluster selection. In this mode, the preferred cluster or node to deploy a service (LTSE, Figure 7a) can be selected.
- Automatic cluster selection, specifying a policy. Now, an application (Kibana, Figure 7b) is deployed considering the cloud cluster with more available resources. In both cases, the previously registered repository of services named “public” was used. The logic and performance of the scheduler depends on the dedicated policy framework selected, mck8s in this case (see [33] for detailed information).

The packaging technology considered (e.g., Helm) allows configuring several parameters at deployment time, modifying a manifest called “values.yaml”. These default parameters can be updated through the form, passing them in the “additional parameters” field.

**Figure 8:**
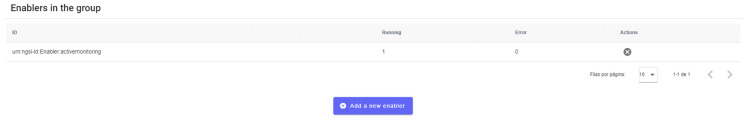
Graphical interface for managing and monitoring the services deployed in a group of dynamic VIMs.

**Figure 9:**
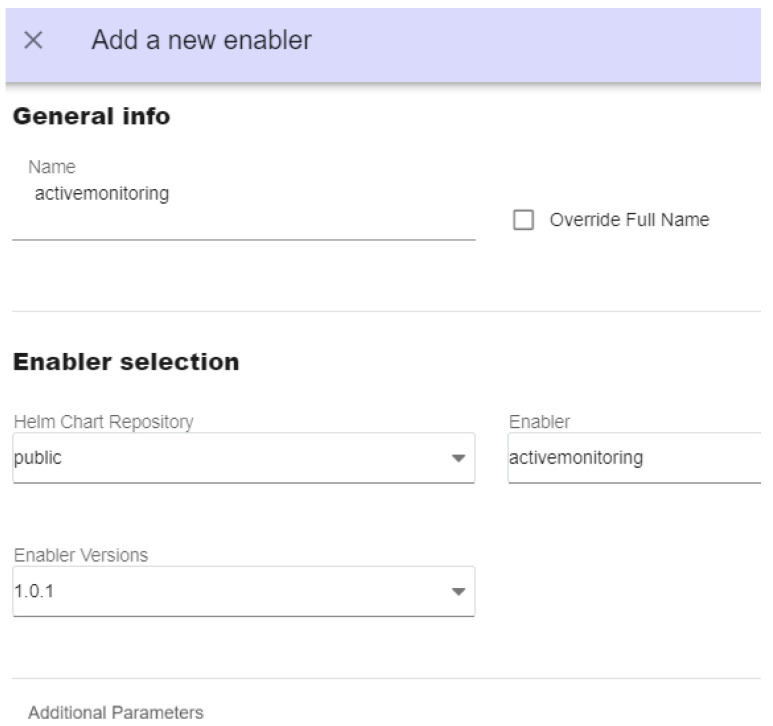
Graphical interface for deploying services in a group of dynamic VIMs.

For the edge services of the use case, there is a specific need of having the same set of services deployed in several edge clusters (i.e., in the whole fleet of vehicles). Given the unfeasibility of having fixed IP addresses in the vehicles and the impossibility of managing their services one by one due to the fleet’s size, their VIMs are locally managed by the orchestrator agents, which in turn is part of a group of the orchestrator, as explained. In this model, any scheduling policy is required (not simply allowed). Some graphical interfaces for managing groups of dynamic VIMs are presented in Figure 8 and Figure 9.

### 3.4. Performance Evaluation

This subsection aims at presenting some figures related to the performance, scalability and error handling of the implemented system. The following aspects have been evaluated: (i) time required by the scheduler for deciding the optimal allocation of a service, based on the number of clusters registered (groups of clusters not included); (ii) API load testing; (iii) scalability of the dynamic clustering (e.g., number of vehicles it could manage); and (iv) handling of errors in the deployment of services over groups of clusters (due to Helm chart downloading and installation errors).

#### 3.4.1. Scheduling Time

The response time required by the scheduler to determine the placement of services in the managed infrastructure was further analyzed using 200 measurements. The response times were recorded using the POSTMAN v11.1.0 tool, and the statistical results demonstrate a high degree of consistency. Specifically, the analysis yields an average response time of 1.687 ms, with a standard deviation of 0.060 ms and following a normal-like distribution (thus, the scheduling time varied between 1.567 and 1.807 ms with a confidence interval of 95%). These values indicate that the response times are tightly clustered around the mean, showing minimal variability, with deviations well within acceptable limits for system performance. In any case, these values were obtained by considering the experimental setup implemented. Factors such as the number of VIMs and groups managed and the performance and availability of the hardware resources would influence the results.

#### 3.4.2. API Load Testing

The purpose is to conduct a load benchmarking to evaluate the performance of the orchestrator’s microservices-based API. The performance testing feature of POSTMAN was used. Its predefined collections and ease of executing tests after making calls, along with the ability to run scripts with specific variables, played a significant role in streamlining and expediting the entire testing process.

The API calls subjected to these tests involved GET, POST, and DELETE methods, which were grouped into three main categories: clusters (i.e., VIMs), repositories, and enablers (i.e., services). The evaluation process begins with executing the POST method, which generates a random cluster with a similarly random name. This approach avoids potential errors that could arise from attempting to create duplicate clusters with identical credentials. The response from this call, which includes the cluster’s unique identifier, is stored in a variable for later use. Next, the GET method is executed to retrieve specific information about the newly created cluster. This step allows validation of the correct creation and availability of the cluster before moving to the next stage of the benchmark. Finally, the DELETE method is measured, using the stored identifier to delete the cluster created in the initial POST call. This deletion phase ensures that the environment is clean and ready for the next test cycle, maintaining the integrity of the load testing process.

**Figure 10:**
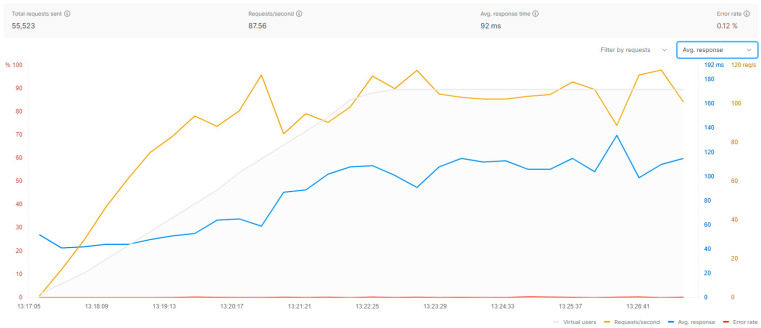
API load testing results.

**Figure 11:**
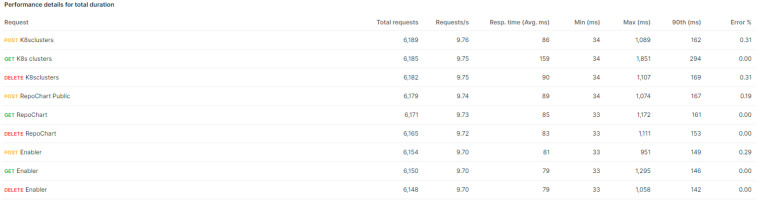
Summary of the API load testing for the different endpoints and methods.

This sequence of calls is applied not only to the clusters but also to the repositories and services during load testing. The cluster for fleet emulate was used for this test (4 CPU cores, 16 GB RAM). Considering these specifications, the load test emulated 60 clients over 10 min. The test does not start with 60 clients but ramps up over a 5 min period until reaching this number. Test metrics are summarized in Figure 10 and Figure 11. While the percentage of errors are low, they occur given the large volume of requests received, which would not be realistic considering typical patterns for registering VIMs (or groups of dynamic VIMs) and repositories and managing services’ lifecycles.

#### 3.4.3. Scalability of the Design for Dynamic VIMs

**Figure 12:**
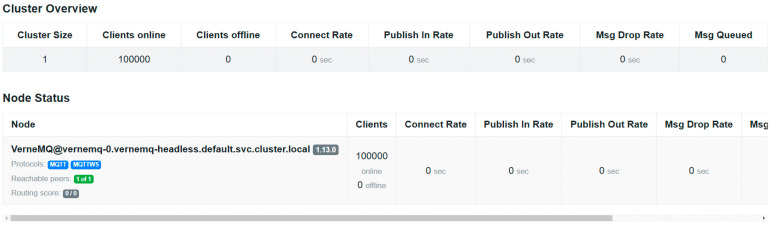
Data broker status webpage. All the emulated clients could be communicated correctly.

Load tests were conducted using the emqtt-bench tool in the fleet emulation cluster, as seen in Figure 12. Although results only reach up to 100,000 clients, it should be highlighted that only one machine was used for hosting a single instance of the cloud’s MQTT data broker. Therefore, it is possible to scale the deployment by increasing the number of replicas and resources, and configuring the data broker so that it can manage this number of clients.

#### 3.4.4. Handling of Errors in Dynamic VIMs

**Figure 13:**
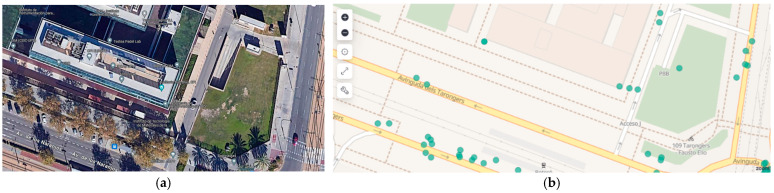
Testing in a scenario without an Internet connection: (**a**) UPV parking area and (**b**) data received. Green dots are real locations of the vehicle capture by its GPS.

Among the tests conducted in a real driving environment at the Polytechnic University of Valencia (UPV) facility, scenarios where the vehicle lacked an Internet connection were prepared. Tests were carried out on the second floor of an underground parking garage, where no connectivity was available. As shown in Figure 13, and owing to GPS data, it can be seen that the vehicle’s data were transmitted the moment the Internet connection was restored (cross-checked with the data available in the local database).

During the tests conducted in the parking garage, the vehicle experienced an average offline period of 15 min. Upon reconnection, all data stored locally were successfully transmitted to the server within 15 s, with no observed data loss or corruption. These results demonstrate the system’s reliability in handling connectivity disruptions.

Additionally, Table 5 presents the performance of the data broker under simulated offline conditions in a controlled laboratory environment. Tests were conducted by simulating message transmission from vehicles to cloud, sending them at a fixed interval of 20 s with varying offline periods (15, 30 and 60 min). The interval was chosen to reflect a typical data reporting frequency for IoT or telematics applications, ensuring a realistic workload for the system during offline and recovery scenarios. This test was repeated 20 times, with identical outcomes despite minor variations in the recovery time.

Results highlight the reliability of the orchestrator in maintaining data integrity, ensuring zero data loss and quick recovery. In case that some data losses had appeared, bottlenecks should be addressed by increasing buffer capacity to handle extended offline periods and adjusting message expiration policies to retain critical data for longer disconnection durations. Overall, the approach proves to be robust for real-world vehicle-to-cloud communication scenarios.

## 4. Discussion

### 4.1. Alternative Edge–Cloud Continuum Orchestration Schemas

The trend towards service orchestration in the edge–cloud continuum paradigm is reflected in the KubeEdge solution. Ongoing since 2018, KubeEdge is an open-source framework based on the Kubernetes architecture that aims to bring all the functionalities offered by K8s to the edge and distributed environments with limited computational resources. This solution keeps the control plane in the cloud, where computational resources are greater, and leaves the application plane workloads at the edge. Its architecture is divided into two components: CloudCore and EdgeCore. CloudCore runs in the cloud environment and is responsible for the centralized management of the K8s cluster and communication with the edge. The EdgeCore component runs on edge devices and functions as a Kubernetes node. KubeEdge provides bidirectional synchronization capabilities between the cloud and edge, capable of restoring the established connection via websockets in case of loss. It also offers support for IoT device connectivity and communication protocols such as MQTT, Modbus and OPC-UA. KubeEdge demonstrated that the framework is capable of orchestrating up to 1 million pods on 100,000 edge nodes, functioning as a single cluster. Although initially it may seem that KubeEdge is a solution more closely related to the presented problem, it should be noted that the present orchestrator solution offers much greater scalability in environments with dynamic IPs and the ability to deploy services in groups. It also provides reliability and resiliency features, as in the case of connection loss or cloud unavailability, a full K8s cluster is in place, with all the set of production-ready features included within it.

Another similar solution is OpenYurt, the first open-source project carried out by the Alibaba group. This is an extension of Kubernetes designed for edge computing environments. It transforms K8s clusters to efficiently operate at the edge, ensuring continuous application management even with intermittent connectivity. OpenYurt implements security by using tunnels that address the connectivity challenges arising from the interaction of different heterogeneous networks to which edge nodes may be connected. Another feature is the NodePool, which groups physical or virtual nodes with similar characteristics and manages them as a unit in an edge computing environment.

KubeVela presents an alternative approach for application delivery and management in distributed architectures. It offers a declarative abstraction based on the Open Application Model (OAM), simplifying the implementation in hybrid and multicluster environments. While not specifically designed for edge computing like the previous ones, KubeVela complements them by providing resilience against connectivity failures as well as progressive deployment strategies. Its ability to integrate and manage services across cloud and edge environments extends its benefits to distributed infrastructures, delivering scalability and flexibility.

Comparing the various strategies, OpenYurt is designed to be minimally invasive, extending K8s functionality with plugins and operators without altering its core structure, while KubeEdge opts for a more radical modification by rewriting certain Kubernetes components, like kubelet and kube-proxy, making it particularly suited for edge data centers or large edge devices with high computational demands. In contrast, KubeVela differs as it operates as an abstraction layer on top of K8s, focusing on application delivery and declarative management model. Unlike OpenYurt and KubeEdge, which are tailored for edge scenarios, KubeVela targets hybrid and multicluster environments, delving into deployment strategies and seamless integration across cloud, on-premises, and edge infrastructures.

Finally, it is also important to monitor and contribute to the advancements carried out in the framework of the computing continuum field, as the IoT ecosystem is in constant evolution. The EUCEI initiative, which homogenizes the outcomes of this research arena, has recently released some initial specifications for the evolution of service and resource orchestration [38]. A key contribution proposed within the framework of the aerOS project is the logical decoupling of the modules related to scheduling and deployment of services, in the so-called “high-level” and “low-level” orchestrators, so that the latter can only perform in their own infrastructure and not in those owned by other stakeholders. While the proposed solution of the present paper already follows this pattern, as deployments are managed by the receiving VIM manager at their respective environments, there are some features that can be of interest for the architecture that are under study. Some of them are the implementation of “adapters” for supporting more types of workloads (e.g., FaaS) and try to make them independent of the underlying hardware and software stacks, the use of distributed registries for high availability and privacy preservation of critical data, and the consideration of common data models and/or ontologies for managing the continuum.

Table 6 summarizes some key differences between the proposed architecture and mentioned relevant existing solutions, including OSM as the reference MANO implementation, in terms of design, scalability, dynamicity and flexibility. As a differentiation, one can observe that features like lifecycle management of a common set of services in large groups of nodes is not foreseen in existing solutions, being prepared for orchestrating both massive edge deployments and traditional cloud environments.

### 4.2. Networking Implementation and Dynamic Clustering

The advantages and disadvantages of the networking solution (implemented here with Cilium) have been evaluated in environments using both static and dynamic IP addresses. While Cilium offers notable benefits in terms of security and overall performance, some analysis regarding the presence of static and dynamic IPs should be made.

The advantages of Cilium in environments with static IPs are first identified. The extended Linux namespace technology and eBPF-based routing employed by Cilium enable exceptionally high performance characterized by low latencies. Also, adopting Cilium significantly enhances security through the implementation of security policies that provide granular access control and robust protection against threats in container environments. Therefore, Cilium copes with NGIoT scalability requirements due to its horizontal scaling capabilities and distributed architecture, enabling efficient management of large workloads and promoting efficient growth of container clusters.

However, certain limitations and challenges were observed when using it. On the one hand, the initial complexity of its configuration was highlighted, which may require additional expertise and effort compared to other solutions available in the market. Cilium was found to be dependent on specific kernel versions, which can limit its portability and require careful operating system updates. Furthermore, considerably higher resource requirements were observed in high-traffic environments, emphasizing the importance of proper planning and hardware sizing when using it. It is also relevant to mention that the Clustermesh feature of Cilium presents configuration challenges and dependency on the underlying network for intercluster traffic routing. These challenges can affect performance in applications requiring low latency and high throughput. On the other hand, in the implementation with dynamic VIMs, the inability to utilize Cilium’s Clustermesh service was observed, due to the lack of fixed, reachable IP addresses from the cloud. This limitation is common to similar solutions.

The advantages and disadvantages of Cilium, as discussed, largely depend on the specific architecture of the environment, and individual considerations of each case need to be taken into account. In future research, the identified challenges with the use of Cilium could be addressed. Improvements in initial configuration, reduction of the dependency on specific kernel versions, optimization of resources, and provision of a common DNS service for the entire cluster framework generated by the Clustermesh service are some lines of potential work. Additionally, exploring the possibility of mitigating intercluster routing challenges by considering different underlying network alternatives could be beneficial. The aforementioned challenges can be extrapolated to alternative existing solutions.

### 4.3. MANO Evolution

MANO was originally considered for telco operators, meaning, VNFs deployed as VMs, rather than for any kind of Cloud Native service or application. The 5G-PPP software network working group already identified all the benefits that Cloud Native MANO implementations could provide in terms of speed, scalability and connectivity [35], together with its role not only for orchestration, but also for observability, service mesh, service discovery, networking, setting up distributed databases, etc. The latter reference also depicted a potential paradigm shift toward a pure Kubernetes-centric MANO architecture. Aligned with this, ETSI NFV has recently updated its specifications to be aligned with this evolution; still, available implementations have work to adapt to it [39], as only basic support is given currently.

This report shows the potential of the MANO framework for orchestrating not just VNFs, but NGIoT workloads in the edge–cloud computing continuum. An architectural example is presented, following MANO principles but not fully aligned with it, for instance, in the dedicated design for dynamic VIMs presented in Section 2. Discussions are needed to decide whether effort should be placed in devising a specification valid for both NFV and non-network services and applications (e.g., NGIoT).

### 4.4. Challenges and Future Research Directions

The proposed orchestration architecture addresses several limitations in existing solutions. The fact of considering the management of massive edge–IoT use cases and being MANO-compatible maximizes its uptake potential in different sections, even in cases where disconnections may occur. It enables implementing resilient over-the-air (OTA) strategies, starting with preselected groups of nodes or clusters for validation and then moving to production ones (the managed fleet, in the presented use case). Still, there are some limitations given the centralized nature of the core components of the orchestrator. As explained in [40], meta-operating systems are emerging as an alternative for managing resources and services in heterogeneous IoT–edge–cloud continuum ecosystems. They consist in a set of core and auxiliary services deployed in all nodes with a minimum computing capabilities, including a much larger number of features and capabilities in comparison to the orchestration agent presented in this paper. Overall, the meta-operating system approach effectively enables the realization of a distributed orchestration solution, facilitating the integration of heterogeneous nodes (in terms of varying resources, virtualization capabilities, containerization technologies, etc.) potentially owned by different stakeholders. Therefore, this alternative design approach could be of greater interest in specific scenarios, however, adding significant complexity in deployment, operation and maintenance.

Future research directions must consider further effort in the definition of interfaces. The architecture foresees the integration of components or frameworks that provides specific features for an architecture implementation. This is the case for scheduling policy frameworks, MANO frameworks, monitoring agents, networking technologies and data brokers. With the exception of the former, which rely on a well-established protocol such as MQTT, the lack of these definitions hinders such integrations, thus requiring tailored efforts. Another interesting interface to consider could be the use of intent-based chatbots powered by dedicated LLM models, with dedicated recommendation models, as the one presented in [41], to support users during their manual operation for increasing their user experience while reducing potential errors.

Furthermore, continuous technological evolution will not stop with the paradigm shift toward the current Cloud Native vision, in which Kubernetes has gradually become the predominant VIM. New research is carried out to maximize the efficiency of the edge–cloud computing continuum, for instance, considering Edge Native applications, WebAssembly (abbreviated Wasm) [42], RISC-V architectures [43] or gRPC interfaces [44]. Also, Edge Native applications were introduced in 2019, and although there are many attributes in common with Cloud Native (e.g., portability, observability, manageability, agnosticism to language and framework support), there are some differences in, e.g., data models, elasticity and scaling in different locations, security and privacy, hardware and location awareness, etc. [45] to consider. In future orchestrator evolutions, dedicated models for declaring edge requirements must be in place so the scheduling component (updated accordingly) can have them in consideration for optimizing its performance, and with the other components (e.g., API, networking, monitoring) adapted accordingly.

On a final note, WebAssembly (Wasm) is a novel technology worth highlighting. Being an open standard from the W3C, the original goal was to enable the execution of high-performance applications on web browsers. However, its design specifications showed great potential for supporting the development of isomorphic IoT applications, including features like modularity, isolation, small footprint and portability [46]. In the virtualization realm, Wasm is becoming a promising alternative to containers, removing barriers related to the execution environment, considering contextual and resource-related information, live migration [47] and, potentially, also reducing processing consumption [40]. Although still immature, the CNCF is already promoting it on its technological landscape and the most popular container engine, Docker, already provides support to it. This entails that the proposed orchestrator architecture already provides support for controlling the lifecycle of services whose components are developed considering WebAssembly; however, further evolutions are needed to fully exploit their advantages.

## 5. Conclusions

This paper has presented an orchestrator architecture for the Next-Generation Internet of Things, based on ETSI MANO and following the trend toward the Cloud Native paradigm. It delves into several aspects, from the scheduling and deployment of services within the managed infrastructure, to their packaging and connectivity. A technological implementation of such architecture and a use case exemplifying its potential is presented, describing its use and measuring its performance.

One of the main challenges of Cloud Native implementations is the reliance on static IP addresses, as necessary for cloud environments. While this might not be problematic in some scenarios, there are situations in which their dynamism and/or scalable nature may require of dedicated approaches. As a key contribution of this article, an alternative workflow is designed for addressing this kind of scenario, considering a publication–subscription pattern that can accommodate resource-constraint computing nodes. This capability has not been observed in existing orchestrator architectures or implementations, and thus could become instrumental in the design of massive, edge–IoT use cases.

Apart from validated in use cases from the automotive vertical like the one presented, the proposed orchestrator has also been tested by actual stakeholders belonging to the construction and logistic sectors. Next steps include its validation in massive 5G–IoT scenarios, to fully exploit its inherent capabilities. Also, large-scale validation of the scheduling and networking policies implemented are required. Finally, further research is needed to design and integrate novel “edge” or “continuum” native technologies on the orchestrator, like WebAssembly, to overcome unresolved challenges and limitations.

## Figures and Tables

**Table 1:** Basic data model for deploying services in dynamic VIMs.

Group	VIM	Service
{ “id”: “groupId”, “type”: “Group”, “description”: { “type”: “String”, “value”: “Group description” }, “services”: { “type”: “array”, “value”: [“serviceName1”, …] }, “dateCreated”: { “value”: “timestamp” }, “dateModified”: { “value”: “timestamp” } }	{ “id”: “vimId”, “type”: “Vim”, “description”: { “type”: “String”, “value”: “VIM description” }, “refGroup”: { “type”: “Relationship”, “object”: “groupId” }, “dateCreated”: { “value”: “timestamp” }, “dateModified”: { “value”: “timestamp” } }	{ “id”: “serviced”, “name”: “serviceName” “type”: “Service”, “refVim”: { “type”: “Relationship”, “object”: “vimId” }, “status”: { “type”: “String”, “value”: “statusValue” }, “info”: { “type”: “String”, “value”: “infoValue” }, “dateCreated”: { “value”: “timestamp” }, “dateModified”: { “value”: “timestamp” } }

**Table 2:** Pub–sub topics for managing the lifecycle of services in dynamic VIMs.

Topic	Message	Description
/fleet/group/id /vim/id	“{op: install, service: serviceName, package: packageName, repository: repositoryURL}”	Service deployment, update or removal (sent from the orchestrator’s API to group or dynamic VIM)
	“{op: update, service: serviceName, package: packageName}” “{op: delete, service: serviceName}”	
/status	“{service: serviceName, op: instantiate, vim: vimId, status: pending, info: pending}”	Service status creation and update (from dynamic VIM to context broker)
	“{service: serviceName “, op: update, vim: vimId, status: running/pending/error, info: running/pending/error}”	

**Table 3:** Technologies leveraged for developing an orchestrator based on the proposed architecture.

Technology	Component/s	Rationale
OSM	NFVO/VNFM	Compliance with ETSI MANO specifications, with support for Cloud Native services. Two versions developed, one without native MANO.
TypeScript/Python	API, packaging, scheduler	TypeScript used as a common programming language to implement custom logic. Python used for the scheduler development.
Go + Helm	Orchestrator agent	Custom logic. Helm as de facto Cloud Native packaging technology.
Cilium	Networking component	CNI plugin covering the requirements posed by the architecture. Also, for compatibility with the framework selected for the scheduling policies.
Prometheus and exporters	Monitoring server, agents	De facto Cloud Native technology for (performance, resources, application) metrics gathering. Exporters used as agents, with local servers per VIM, and federated version for the orchestrator’s cluster.
Context broker	FIWARE’s Orion	Open-source technology used for managing contextual data. Widely promoted by the European Commission for developing smart solutions.
mck8s + K8s’ HPA + Neural Prophet	Policy frameworks	mck8s considered as it implements some interesting scheduling policies (i.e., most resources, fitting resources, most traffic, see [33]) aligned with the architecture. Features extended with NeuralProphet [34] and K8s HPA, to manage the replicas of the services replicas based on usage forecasts.
Kafka (orchestrator), RedPanda (agents)	Communication bus	Widely used and supported technology for ensuring scalability of communications when the number of services and volume of data increases.
ASSIST-IoT’s LTSE	BBDD	ASSIST-IoT service that provides access to either or both SQL and NoSQL (Elasticsearch) databases with secured API.
ASSIST-IoT’s EDB	Data brokers	ASSIST-IoT service that extends MQTT technology with filters and rule engine.
HAProxy	Load balancer	State-of-the-art technology for balancing the load among data broker instances.
K8s, K3s	VIM	De facto container orchestration system technology. Encouraged by 5G-PPP [35], tested with open-source distributions.

**Table 4:** Resources of the computing infrastructure utilized in the experimental setup.

Cluster/Device	K8s Nodes	CPU Cores	RAM	Storage
Cluster 1 (K8s)	Master	6	32 GB	84 GB
	Worker-1	4	8 GB	32 GB
	Worker-2	4	8 GB	128 GB
	Worker-3	4	8 GB	128 GB
Clusters 2-5 (K8s)	Master	6	16 GB	32 GB
Fleet emulator (K8s)	Master	4	16 GB	128 GB
Vehicle (K3s)	Master	4	8 GB	32 GB

**Table 5:** Data recovery statistics after network disruptions.

Offline Duration	Data Lost During Offline	Data Recovery Success	Average Recovery Time
15 min	0%	100%	7 s
30 min	0%	100%	5 s
1 h	0%	100%	10 s

**Table 6:** Relevant differences between existing solutions and the proposed orchestration architecture.

Solution	Design	Scalability	Dynamicity	Deployment Flexibility
OSM MANO	On top of K8s. It interacts with clusters and manage services lifecycle on them	To be installed in a K8s cluster that acts as the control plane, which is capable of handling static K8s clusters	Limited, as previously created K8s clusters must be reachable through a public IP	Services can be deployed in manually selected K8s clusters, with YAML manifests, Juju charms and Helm charts. It does not provide built-in capabilities for bulk service deployments in large (geographical, logical) groups of edge nodes
KubeVela	On top of K8s. It interacts with clusters and manage services lifecycle on them	To be installed in a K8s cluster acting as control plane. Capable of handling several static K8s clusters and standalone edge nodes	Limited, as previously created K8s clusters and standalone nodes must be reachable by the control plane through a public IP	Services can be defined in both K8s-based resources (manifests and Helm charts) and custom definitions. It also provides multicluster deployments, GitOps support using FluxCD and add-ons for day-2 operations after deployments
KubeEdge	Re-design of K8s for edge computing environments. Divided into cloud and edge components. De facto CNCF K8s distribution for edge computing	It has proved its vast scalability in a use case in which more than 100,000 edge nodes were deployed across the Hong Kong–Zhuhai–Macao bridge	High, as it has been designed specifically to manage edge computing nodes with constrained resources, dynamic IPs (even behind LANs) and with unreliable network conditions	Same as a regular K8s cluster but adding some functionalities on top, e.g., to interconnect deployed services in heterogeneous edge nodes using a custom service mesh. It does not provide built-in capabilities for bulk service deployments in large groups of edge nodes
OpenYurt	Enhancement of K8s through custom plugins and operators	A single cloud node is capable of managing vast volume of edge nodes	High, as it has been designed specifically to manage edge computing nodes. Still, edge nodes must have higher computational requirements and more reliable and trusted network conditions	Definition of node-pools to split deployments in different regions. It also provides a custom edge–cloud service mesh implementation
This study	On top of K8s. It interacts with clusters and manage services lifecycle on them	Highly scalable as it allows adding more than 200,000 clusters and managing them as fleets or as individual elements if they have dynamic IPs	High dynamicity, as it was designed to manage edge computing nodes with dynamic, private IPs. It is compatible with any lightweight Kubernetes distribution, such as K3s	It offers the ability to deploy Helm charts without considering the type of cluster. For static clusters, it automatically creates the Cilium clustermesh and allows edge-to-cloud services to connect via DNS names and in an encrypted manner. It also allows deploying Helm charts to a fleet of vehicles or to a single unit

## Data Availability

Data are contained within the article. System implementation of the proposed architecture can be found at [32].
